# Hydatid Cyst of the Adrenal Gland: Is Radical Surgery Necessary for Recurrent Hydatid Disease?

**DOI:** 10.1155/2018/6452402

**Published:** 2018-02-22

**Authors:** Sami Akbulut, Mehmet Yilmaz

**Affiliations:** ^1^Department of Surgery and Liver Transplant Institute, Inonu University Faculty of Medicine, 44280 Malatya, Turkey; ^2^Department of Surgery, Inonu University Faculty of Medicine, 44280 Malatya, Turkey

## Abstract

Hydatid cyst disease caused by *E. granulosus* is a zoonotic disease that may involve many body tissues and organs, mainly liver. Adrenal glands are rarely involved even in regions where hydatid cyst disease is endemic. A limited number of studies have been reported on adrenal gland involvement by hydatid cyst disease. Herein, we aimed to report a recurrent case of adrenal hydatid cyst that was misdiagnosed as a hepatic hydatid cyst. A 16-year-old boy with a history of partial cyst excision and evacuation for perforated adrenal hydatid cyst disease three years ago presented to our outpatient clinic with nonspecific abdominal pain. Ultrasonography and computed tomography showed a lesion with an approximate size of 70 × 70 mm compatible with a hydatid cyst, which originated from the segment 5-6 of the liver and extended to the posterior-inferior direction. A surgical intervention was scheduled after a 2-week prophylactic albendazole treatment. During laparotomy using the old incision, it was noted that the cystic lesion that reportedly located in liver was actually a recurrent right adrenal cyst. As dense adhesions existed, the cystic lesion was excised en bloc with the right adrenal gland. As the lesion was totally removed without getting ruptured, albendazole was not administered at the postoperative period. No recurrence was observed at one-year follow-up.

## 1. Introduction

Despite four different parasites belonging to the echinococcal species having been discovered to cause echinococcal disease in humans, *E. granulosus* is responsible for approximately 95% of all echinococcal cases. Also known as cystic echinococcus, hydatid cyst disease most commonly involves the liver, lungs, spleen, and kidneys [1, 2]. Adrenal glands are one of the most rarely involved organs even in regions where the disease is endemic [2, 3]. Most patients with adrenal hydatid cyst disease (AHCD) may remain asymptomatic for years, and the majority of cases are diagnosed incidentally by radiological studies performed for other indications [2, 4]. A smaller portion of patients become symptomatic as a result of the size, localization, adjacent organ compression, and complications (fistula, perforation, etc.) of a cyst [2]. Hydatid cyst disease is treated with one or several of the treatment options including medical treatment, surgical treatment (open or laparoscopic), percutaneous treatment by different techniques such as the puncture-aspiration-injection-reaspiration (PAIR) method, standard catheterization technique or modified catheterization technique (MoCaT), and watch and wait method [2, 5]. It is still unclear which surgical treatment option is better among several options including partial cystectomy or en bloc resection together with the adrenal gland. Herein, we aimed to report our clinical management approach to a recurrent case of AHC that was previously misdiagnosed as a hepatic hydatid cyst by radiological studies.

## 2. Case Report

**Figure 1:**
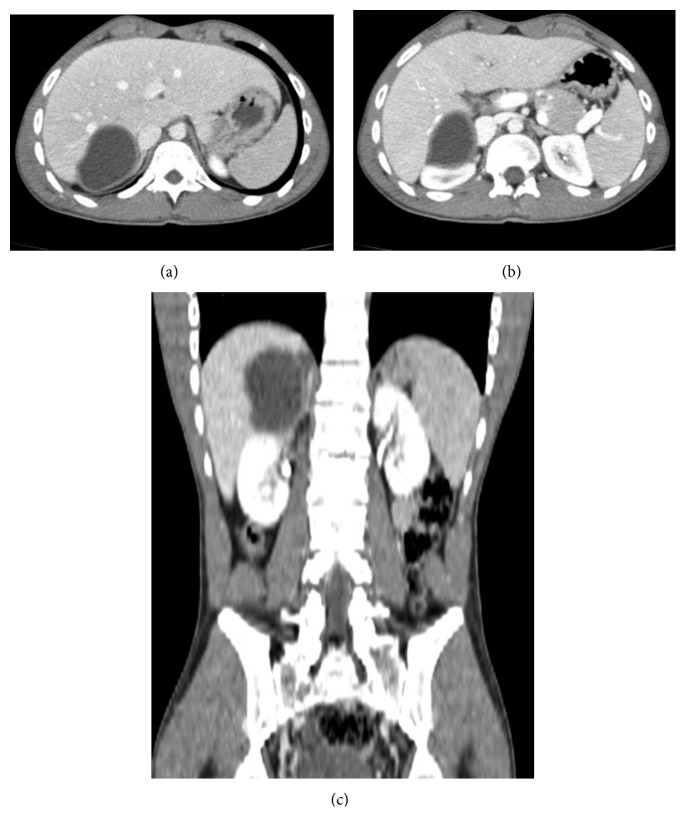
Axial (a, b) and coronal (c) contrast-enhanced CT images. A lesion with lobulated contours and a size of 70 × 70 mm that was compatible with a hydatid cyst. The lesion originates from the hepatic segment V-VI and gives off exophytic extensions in the posterior-inferior direction.

A 16-year-old boy presented to our outpatient clinic with upper right quadrant pain. He explained that he had presented to our hospital's pediatric surgery clinic with skin redness, impaired general status, and cyanosis three years earlier, when a perforated AHC was diagnosed in radiological studies and treated surgically. Upon review of his previous medical reports, it was noted that the contents of the cyst in the right adrenal gland were removed, followed by the resection part of the cyst's anterior wall and the irrigation of the abdominal space with 10% polyvinylpyrrolidone iodine solution. He stated that he had always resided at a city center and had no animal contact ever before. On physical examination, no abnormality was noted apart from a right-sided subcostal incision. His hemogram and liver and kidney function tests were within normal limits. His serological tests revealed an ELISA IgG: 15.66 (positive). A contrast-enhanced computed tomography showed a lesion with lobulated contours and a size of 70 × 70 mm that was compatible with a hydatid cyst. The lesion originated from the hepatic segment V-VI and gave off exophytic extensions in the posterior-inferior direction, and it contained hypodense areas (daughter vesicles) (Figures 1(a)–1(c)). The patient was operated on after a 2-week prophylactic albendazole treatment at a dose of 400 mg b.i.d. The old subcostal incision was used to access the abdominal cavity. It was noted during the exploration that the cystic lesion that reportedly located in the liver actually originated from the right adrenal gland and externally compressed the inferior vena cava. After dissecting adhesions between the right lobe of the liver and the cystic lesion, the location of the cystic lesion and its right adrenal origin were revealed. Since dense adhesions between the cyst and surrounding tissues did not permit excision of the cyst in isolation and since the disease had recurred, the cystic lesion was excised en bloc with the right adrenal gland, without its integrity being disrupted. The patient was then discharged without any problem. Albendazole was not postoperatively administered due to total excision of the lesion. No recurrence was observed at one-year follow-up.

## 3. Discussion

Cystic lesions of the adrenal glands are extremely rare, with the majority being incidentally discovered at autopsy series or detected by radiological studies. Therefore, their actual incidence is unknown although some series have provided incidence values of 0.06–0.18% [6]. The most common nonneoplastic adrenal cysts are endothelial cysts, pseudocysts, epithelial cysts, and parasitic cysts (6-7%) [4, 6–8]. Hydatid cysts are the main parasitic cysts involving the adrenal glands [2]. AHC is unilateral in 90% of cases [9].

Adrenal glands are one of the organs that are most rarely involved by hydatid cyst disease even in regions where the disease is endemic. AHC disease constitutes for less than 1% of all hydatid cyst cases [4–12]. The disease usually occurs secondary to disseminated hydatid disease [10, 11]. Isolated AHC disease is relatively rare, with only a limited number of cases being reported in the literature [9, 10]. As is the case for other hydatid cysts involving other organs, AHC is usually asymptomatic, usually being detected incidentally in radiological studies performed for other indications [4, 9, 12]. AHCD rarely becomes symptomatic, with most symptoms being related to the interaction of a cyst with adjacent organs and tissues (e.g., compression and inflammation) [3, 4, 9, 12]. Particularly, inflammation causes signs and symptoms of peritoneal irritation [9]. The most common symptoms are related to the gastrointestinal systems, such as flank pain, sense of fullness, constipation, and loss of appetite [9, 11]. The most serious complications associated with the AHC disease is cyst rupture leading to anaphylaxis and hemorrhage [1]. Arterial hypertension named as the Goldblatt phenomenon may develop as a result of external compression of a renal artery or irritation of functional gland parenchyma by large cysts [2, 4, 9, 12]. Signs and symptoms such as headache, palpitations, and hypertension resembling a pheochromocytoma may also be seen as a result of compression of adrenal medulla by cysts that grow into the adrenal gland [9]. Despite rare, various symptoms may develop due to the fistulization of AHCD into adjacent intestinal hollow organs [11].

Both definitive and differential diagnosis of the AHCD can be made by a combination of patient's history, blood tests for functional adrenal lesions, radiological studies, and serological tests [1, 2, 13]. Radiological instruments such as US, CT, and MRI usually provide enough information for differential diagnosis. Serological tests do not have a prominent importance for the diagnosis of cystic echinococcosis as positive serological tests do not confirm the diagnosis and negative serological tests do not exclude it. Some serological tests are often used in follow-up to detect recurrence disease. One of the two existing studies using 18F-FDG PET/CT to diagnose AHCD showed no significant FDG uptake, whereas the other demonstrated an increased FDG uptake [2, 13]. Although 18F-FDG PET/CT has no role in the diagnosis of AHCD, the popularity of PET/CT for the differential diagnosis of tumors of this region continues to increase.

The general principles of AHCD treatment are the same as those for other organ hydatid cyst diseases. To summarize, the most important parameters used for the management of AHCD are the size and the localization of a cyst, and presence of complications. However, several factors such as the adrenal gland being small, destruction of the adrenal gland by the cyst, and the state of the contralateral adrenal gland determine which treatment modality to apply. The most commonly performed modality for AHCD is total or partial excision of cystic lesion and the administration of pre/postoperative medical treatment. Medical treatment is not needed for cases where the cystic lesion is totally excised. Surgical treatment can be applied by open or laparoscopic approach depending on the surgical expertise of a given center [3, 6]. In parallel to technological advances, transperitoneal and retroperitoneal laparoscopic surgery have emerged as good alternatives [3, 4, 6].

There exist only two reports of the use of the PAIR method for AHCD [7, 8]. Akhan et al. [8] reported the advantages of the PAIR method as a limited contamination and preservation of the adrenal gland. In contrast, some authors have advocated that the PAIR method should be avoided in cases with AHCD [14]. To our opinion, this prejudice mainly stems from the concern of an anaphylactic reaction and spilling of the cyst contents over through a defect formed by the puncture needles during the procedure. Nevertheless, this risk is trivial, as the puncture needle passes through the parenchyma of the parenchymal organs such as the liver, kidneys, and spleen. The other concern, albeit to a lesser importance, is the possibility of the cystic lesion being a pheochromocytoma. We opine that PAIR is feasible when applied by experienced radiologists under the coverage of neoadjuvant albendazole treatment for early stage AHCD that are well differentiated from other lesions. Hence, Akhan et al. [8], who had a significant expertise in PAIR, reported that in patients with AHCD successful outcomes can be achieved using PAIR provided that the basic principles of the technique are complied with. Another method for patients with AHCD is the watch and wait method, which suits for uncomplicated WHO Stage VCE4 and CE5 cysts.

In cases with an indication for surgical treatment, the treatment modality to be applied should be selected. Whether open or laparoscopic, there are two surgical treatment approaches: radical (pericystectomy and en bloc adrenalectomy with cystectomy) and conservative (partial cystectomy). Some authors advocate that AHCs usually reach a large size, destroying the adrenal gland during their growth. Hence, they recommend en bloc resection of the adrenal gland together with the cyst [4, 6, 11, 12]. Some authors suggest that it is usually impossible to reach a surgical plane between the cyst wall and gland parenchyma, and one of the options of en block resection or gland-preserving partial cystectomy should be attempted whenever pericystectomy is not feasible [10]. To our opinion, irrespective of the surgical method used, an adrenal gland-preserving resection (pericystectomy and partial cystectomy) should be primarily attempted for this benign disease. In cases with dense adhesions not permitting gland-preserving surgery, or in cases that recur despite conservative surgery, en block adrenalectomy with cystectomy can be performed provided that the contralateral adrenal gland is functional.
